# Assessment of perioperative minute ventilation in obese versus non-obese patients with a non-invasive respiratory volume monitor

**DOI:** 10.1186/s12871-017-0352-0

**Published:** 2017-04-26

**Authors:** Jaideep H. Mehta, Davide Cattano, Jordan B. Brayanov, Edward E. George

**Affiliations:** 1 University of Texas Medical School at Houston, 6431 Fannin Street, Houston, TX 77030 USA; 2 Respiratory Motion Inc., 411 Waverley Oaks Rd #150, Waltham, MA 02452 USA; 3 Massachusetts General Hospital, Harvard Medical School, 55 Fruit St, Boston, MA 02114 USA

**Keywords:** Respiratory monitoring, Respiratory requirements, Minute ventilation, Perioperative safety, Opioids, Obesity

## Abstract

**Background:**

Monitoring the adequacy of spontaneous breathing is a major patient safety concern in the post-operative setting. Monitoring is particularly important for obese patients, who are at a higher risk for post-surgical respiratory complications and often have increased metabolic demand due to excess weight. Here we used a novel, noninvasive Respiratory Volume Monitor (RVM) to monitor ventilation in both obese and non-obese orthopedic patients throughout their perioperative course, in order to develop better monitoring strategies.

**Methods:**

We collected respiratory data from 62 orthopedic patients undergoing elective joint replacement surgery under general anesthesia using a bio-impedance based RVM with an electrode PadSet placed on the thorax. Patients were stratified into obese (BMI ≥ 30) and non-obese cohorts and minute ventilation (MV) at various perioperative time points was compared against each patient’s predicted minute ventilation (MV_PRED_) based on ideal body weight (IBW) and body surface area (BSA). The distributions of MV measurements were also compared across obese and non-obese cohorts.

**Results:**

Obese patients had higher MV than the non-obese patients before, during, and after surgery. Measured MV of obese patients was significantly higher than their MV_PRED_ from IBW formulas, with BSA-based MV_PRED_ being a closer estimate. Obese patients also had greater variability in MV post-operatively when treated with standard opioid dosing.

**Conclusions:**

Our study demonstrated that obese patients have greater variability in ventilation post-operatively when treated with standard opioid doses, and despite overall higher ventilation, many of them are still at risk for hypoventilation. BSA-based MV_PRED_ formulas may be more appropriate than IBW-based ones when estimating the respiratory demand of obese patients. The RVM allows for the continuous and non-invasive assessment of respiratory function in both obese and non-obese patients.

## Background

Adequate respiratory monitoring is necessary for patients undergoing surgery, and is especially crucial in the post-operative period where the patient’s airway is not secured and staff presence is intermittent. Several factors can lead to respiratory compromise and respiratory depression, with the use of sedatives and opioids being a prominent example. Obstructive breathing patterns, often unappreciated preoperatively, can be initiated or exacerbated by anesthetics and opioids and trigger postoperative apnea (POA). It has been repeatedly shown that patient controlled analgesia (PCA), which was thought to be a safe alternative to staff-administered analgesia, has not eliminated opioid induced respiratory depression (OIRD) which remains one of the most dangerous adverse post-operative outcomes [1, 2].

Postoperative apnea (POA), unlike other forms of apnea, is often a combination of both obstructive and central apnea [3]. The effect of opioids on respiratory drive results from both direct depressant action on respiratory neural activity as well as from the reduction in pharyngeal muscle tone. Obese patients account for up to 58% of adult obstructive sleep apnea (OSA) cases and a 10% increase in body mass leads to a six-fold increase in the risk of developing moderate to severe OSA [4]. Obstructive respiratory patterns, postoperative pulmonary atelectasis, [5] reduced functional residual capacity, reduced vital capacity, and increased metabolic (and respiratory) demand all lead to increased perioperative risk in obese patients [5–9]. With these risk factors in mind, respiratory monitoring and timely identification of respiratory insufficiency in the perioperative period is paramount for obese patients.

Indirect respiratory monitoring like oxygen saturation (SpO_2_) and capnography (EtCO_2_) are unable to identify early signs of respiratory compromise and can delay interventions [10]. Moreover, both SpO_2_ and EtCO_2_ monitoring systems are plagued by false alarms in the post-operative setting [11, 12]. To address these issues, a non-invasive respiratory volume monitor (RVM) providing direct, real-time measures of minute ventilation (MV), tidal volume (TV) and respiratory rate (RR) in non-intubated patients has been developed.

Previous research shows that the RVM can reliably provide accurate real-time continuous respiratory volume measurements when compared against a monitoring spirometer or a ventilator, with approximately 90% accuracy for MV and TV, and 98% accuracy for RR [13, 14]. The RVM applies a small amount of high-frequency electrical current to the patient’s thorax and monitors changes in the electrical signal using three EKG-like electrodes placed at the sternal notch, xiphoid and right mid-axillary line. The RVM uses the difference in conductivity between air and tissue to calculate the amount of air moving in and out of the lung in real time and displays an easily interpretable breath-by-breath waveform as well as 30-second averages for MV, TV and RR.

This RVM has also been shown to help identify “true” desaturation events, which can help reduce the rate of false SpO2 alarms due to patient movement and other variables. This is particularly useful in more mobile patients recovering from surgery [15]. In addition, changes in MV have been shown to reflect the patient’s actual respiratory status quicker and with better fidelity than conventional EtCO2 monitoring [16, 17].

In this study we used the RVM to monitor respiratory status in obese and non-obese orthopedic patients and compare ventilation between these two patient categories throughout their perioperative course. We also compared measured MV to known standards of predicted MV in an attempt to better understand the differences in metabolic (respiratory) demand amongst the obese patients.

## Methods

This study was conducted at the Massachusetts General Hospital (MGH) and the Partners Institutional Review Board approved its design (2011P002898, Boston, MA). All patients provided written informed consent prior to enrollment. Inclusion criteria were English-speaking men and women aged 18 to 99 years undergoing elective joint replacement surgery. There were no specific exclusion criteria. Here, we specifically selected patients undergoing surgery under general anesthesia so that we could have data as to the ventilator settings used during surgery.

We used a bio-impedance based RVM (ExSpiron 1Xi, Respiratory Motion, Waltham, MA) to collect digital respiratory data via an electrode PadSet placed on the thorax as previously described (See Fig. 1 in Voscopoulos et al., 2014^3^). We also collected pertinent demographic, physiologic, and clinical data. The RVM and physiologic data were collected beginning in pre-operative holding, throughout the surgical procedure, and ending post-operatively following sign-out from the post-anesthesia care unit (PACU). Note that, all clinical staff involved in the patient care (surgery, anesthesia, PACU staff, etc.…) were blinded to the RVM measurements. As such, the RVM was not used to inform or alter patient care, and was only used to collect respiratory data for research.

**Fig. 1:**
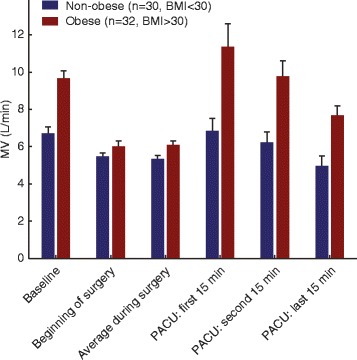
Comparison of MV measurements in obese (Red, BMI ≥ 30) and non-obese (Blue, BMI < 30) patients at various points throughout their perioperative course. The measured MV in the obese population was significantly higher at every time point throughout the course: pre-operatively (Baseline, 9.7 ± 0.4 vs. 6.7 ± 0.3 L/min), beginning of surgery (6.0 ± 0.3 vs. 5.5 ± 0.2 L/min), during surgery (6.1 ± 0.2 vs. 5.3 ± 0.2), on arrival at the PACU (first 15 min: 11.4 ± 1.2 vs. 6.8 ± 0.7 L/min and second 15 min: 9.7 ± 0.8 vs. 6.2 ± 0.6 L/min) and at discharge (last 15 min: 7.7 ± 0.5 vs. 5.0 ± 0.6 L/min). These results are incongruent with IBW-based predicted MV (MV_PRED_) which were very similar (6.2 ± 0.2 vs. 6.1 ± 0.2 L/min, *p* = 0.37) and more closely aligned with BSA-based MV_PRED_ (7.8 ± 0.2 vs. 6.9 ± 0.2 L/min, *p* = 0.0002)

During post-hoc analysis of the data, patients were stratified according to obesity status (BMI ≥ 30 kg/m^2^) and observed MV was compared against the patient’s predicted MV (MV_PRED_) based on both ideal body weight (IBW) and body surface area (BSA) formulas [18, 19]. Ventilator MV (Apollo, Draeger, Telford, PA) was also collected during general anesthesia and was assumed to be “adequate” as it was adjusted to maintain appropriate EtCO_2_ during surgery. The number of opioid doses (either 1 mg morphine or 0.2 mg hydromorphone) as well as the total morphine equivalent of opioids received in the PACU were compared across the two cohorts. We used a conversion factor of 0.16 mg hydromorphone = 1 mg morphine equivalent (MME) and 1 mg of morphine = 1 MME [20].

The main goal of this study was to assess and report on any differences in respiratory volumes between obese and non-obese patients. The secondary goals of the study assessed whether (a) predicted MV formulas based on IBW or BSA would more adequately capture the actual MV observed in the 2 patient cohorts and (b) differences in opioid use in the PACU could explain any potential differences in MV.

Unpaired two-tailed t-tests were used to compare the MV measurements and opioid dosing between the two patient subgroups (non-obese vs. obese) at several time points of the study. Specifically, comparisons were made pre-operatively (Baseline), at the beginning of surgery, during surgery, the first and second 15 min upon arrival at the PACU, and the last 15 min prior to discharge. Two-sample F-tests for equal variance were used to compare distributions of MV at these same time points. Since we had collected multiple [repeated] measurements from each patient in this study, we used a more conservative p < 0.01 cutoff to evaluate the significance of our findings in order to account for any effects of auto-correlation amongst within-patient measurements. All analyses were performed in Matlab 2012b.

## Results

In this study we enrolled 62 orthopedic patients (Age: 65.4 ± 12.0 years, BMI: 30.8 ± 6.0 kg/m^2^), 32 of them were classified as obese and 30 as non-obese. Both the obese and non-obese cohorts had similar MV_PRED_ based on ideal body weight (IBW): Obese: 6.2 ± 0.2; Non-Obese: 6.1 ± 0.2 L/min, p > 0.3, since IBW-based MV_PRED_ formulas are entirely based on patient’s height and sex and not their weight. However, obese patients were managed during surgery at a significantly higher MV while intubated (6.1 ± 0.2 vs. 5.3 ± 0.2 L/min, *p* = 0.0068), suggesting that IBW-based MV_PRED_ may not adequately capture the increase in metabolic demand in obese patients.

If instead we use MV_PRED_ calculated based on each patient’s Body Surface Area (BSA), we see that MV_PRED_ for the obese population is significantly higher than the non-obese (7.8 ± 0.2 vs. 6.9 ± 0.2 L/min, p = 0.0002). Using BSA instead of IBW increased MV_PRED_ in the obese by more than 25%, while the increase in the non-obese was just 11%, suggesting that IBW or BSA -based MV_PRED_ are similar in non-obese patients, but differ substantially in the obese.

During periods of spontaneous breathing perioperatively, the differences in MV between the two cohorts were even more significant, as summarized in Fig. 1. Obese patients systematically had higher MV than non-obese patients pre-operatively (9.7 ± 0.4 vs. 6.7 ± 0.3 L/min, p = 0.0000003), at PACU arrival (11.4 ± 1.2; 6.8 ± 0.7 L/min, p = 0.0014), and at PACU discharge (7.7 ± 0.5; 5.0 ± 0.6 L/min, *p* = 0.0004). All of these differences were significant at a level of *p* < 0.01. Note that, 11 of the 62 patients here (17.7%) had a previous diagnosis of OSA, 9 of these patients with known OSA were obese (28.1% of the obese cohort) and 2 were non-obese (6.7% of the non-obese cohort). None of these patients with known OSA received any specific therapy or treatment related to their OSA in the PACU.

**Fig. 2:**
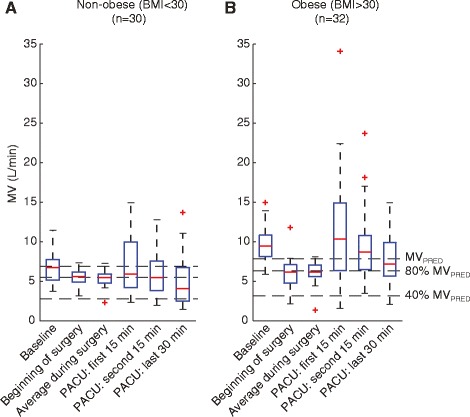
Patient-to-patient variability in minute ventilation (MV) in non-obese and obese orthopedic patients at different time-points in the perioperative course. Boxplots show MV mean and variance in (**a**) non-obese and (**b**) obese orthopedic patient cohorts at seven time-points pre-operatively, intra-operatively, and post-operatively. Dashed lines depict BSA-based MV_PRED_, as well as at-risk (80% MV_PRED_) and un-safe (40% MV_PRED_) ventilation thresholds. Post-operative MV variance is significantly greater in obese patients than in non-obese patients (*p* < 0.01), particularly in the PACU, and whereas the average MV at PACU discharge in the obese patients is significantly higher than in the non-obese patients, 10% of the obese patients spent >1/3 of the last half-hour in the PACU with un-safe MV

**Fig. 3:**
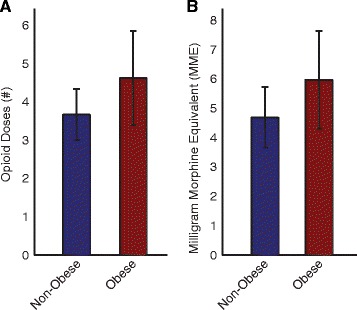
Opioid usage in the PACU in non-obese (*blue*) and obese (*red*) orthopedic patients. (**a**) Average number of opioid doses administered over the course of a PACU stay in the obese and non-obese orthopedic patient cohort, respectively. (**b**) Average total morphine equivalent dose of opioids administered in the PACU. Error bars represent the standard error of the mean (SEM). There is no significant difference observed in PACU opioid dosage when patients are stratified for obesity (*p* > 0.05 for both comparisons)

However, while obese patients had generally higher MV than the non-obese, a substantial fraction of obese patients experienced respiratory depression in the PACU (see Fig. 2). In fact, 10% of the obese patients spent more than 1/3 of their last 30 min in the PACU with un-safe MV (MV < 40% MV_PRED_) [3]. In the non-obese population, the fraction of patients with Low MV for >1/3 of the last 30 min was substantially higher (37%), which could be due to the similar dosing of opioids in both groups despite differences in patient body mass. As Fig. 3 shows, there was no significant difference in the number of PCA opioid doses administered to the obese vs. non-obese group (3.7 ± 0.7 vs. 4.7 ± 1.2 doses, *p* > 0.05, Fig. 3a) or the total morphine equivalence of opioids administered in the PACU (4.7 ± 1.0 vs. 6.0 ± 1.6 MME, *p* > 0.05, Fig. 3b).

## Discussion

We noted significant differences in MV across the two cohorts, with the obese patients systematically sustaining higher MV. This is at odds with the predicted MV (MV_PRED_) based on IBW and much more consistent with MV_PRED_ based on BSA. Additionally, MV measurements for obese patients pre- and post-operatively (while spontaneously breathing and not intubated) were often significantly higher than while intubated and on the ventilator. Yet a significant fraction of the obese patients suffered transient respiratory depression (episodes of MV < 40% MV_PRED_) while treated with standard-dose opioids.

This is the first study to compare the perioperative respiratory demand (in the form of minute ventilation) between obese and non-obese patients, which only became possible since the development and clinical implementation of a non-invasive respiratory volume monitor. According to a recent study, [16] EtCO_2_ measurements could not adequately characterize rapid changes in ventilation either with an in-line sensor, or with an oral/nasal cannula. Several previous studies have shown that the RVM is a more accurate and reliable monitoring option than alternative monitoring technologies such as capnography [13, 14]. This is particularly important in procedural sedation and non-operating room anesthesia, where EtCO2 and oxygen saturation are usually the only available monitoring modalities for respiratory insufficiency, yet are often unreliable or delayed [17].

Since the accuracy of the RVM measurements has been previously demonstrated, the RVM now can provide useful clinical data implications to improve assessment and treatment in specific patient populations The objective of this work was to assess respiratory parameters in non-intubated obese versus non-obese patients, which had not previously been possible. Although it will be important to extend this work to more specific evaluations, here we did not analyze the effects of intra-operative opioids, paralytics, and anesthetic agents and we did not stratify patients based on their other comorbidities or confounding factors. A limitation to the study is that uniformly recorded pain scores were not available from the PACU. While potentially interesting, EtCO2 measurements were not part of standard clinical care and were not obtained during this study. Additional work is needed to help extend these preliminary findings to optimize clinical practice.

A distinct difference in the patient-to-patient respiratory variability became evident between the obese and non-obese cohorts (Fig. 2). Pre-operatively both patient cohorts exhibited similar patient-to-patient variability in MV (i.e. the distribution of MV measurements across patients had similar variance), whereas in the post-operative period the patient-to-patient variability in the obese cohort was significantly larger. This suggests that some obese patients, despite generally having higher minute ventilation, experience episodes of hypoventilation in the PACU associated with unpredictable swings in MV. By monitoring changes in both respiratory rate and tidal volume, individualized treatment plans could be implemented for obese patients.

Specifically in the obese population, Schumann et al. [21] previously suggested that traditional IBW-based MV nomograms may be suboptimal in the defining Predicted MV. Our findings support that conclusion and suggest the preferential use of BSA-based formulas. In this study, the PACU opioid dosage was not significantly different between the obese and non-obese patients, suggesting the differences observed in MV across the two groups cannot be attributed to a greater opioid dosage.

This study demonstrated the utility of the RVM in defining baseline respiratory characteristics in obese vs. non-obese patients. Importantly, real-time respiratory monitoring could help define each individual patient’s respiratory demand and enable individualized care. This could enable deeper understanding of the underlying physiology and help prevent respiratory depression and improve patient safety.

## Conclusions

Our study demonstrated that obese patients have greater variability in ventilation post-operatively when treated with standard opioid doses, and despite overall higher ventilation, many of them are still at risk for hypoventilation. The conventional IBW-based MV_PRED_ formulas may not be accurate predictors of expected respiratory performance in obese patients. Instead, BSA-based MV_PRED_ formulas appear to capture the baseline function and real-time changes in metabolic demand in the obese population more accurately. In addition, the use of the RVM may allow for the continuous and non-invasive assessment of respiratory function, provide more clinically useful data than either EtCO2 or SpO2, and assist in the adjustment of treatment to individualize care in both obese and non-obese patients.

## Abbreviations

BSA: Body surface area
EtCO_2_: End-tidal carbon dioxide
IBW: Ideal body weight
MME: Morphine-milligram equivalent
MV: Minute ventilation
MV_PRED_: Predicted minute ventilation
PACU: Post-anesthesia care unit
PCA: Patient-controlled analgesia
RR: Respiratory rate
RVM: Respiratory volume monitor
TV: Tidal volume.
